# MDA-MB-231 cell morphology influences chemotactic sensing of CXCL12 gradients in type 1 bovine collagen matrix

**DOI:** 10.1371/journal.pone.0343188

**Published:** 2026-07-08

**Authors:** Marsophia Marcellus, Catherine J. Murphy

**Affiliations:** Department of Chemistry, University of Illinois Urbana-Champaign, Urbana, Illinois, United States of America; University of Arizona, College of Medicine-Phoenix, UNITED STATES OF AMERICA

## Abstract

Chemotaxis plays a critical role in the metastatic progression of breast cancer. The chemokine CXCL12 is well recognized as an essential component of chemotactic migration in triple-negative breast cancer (TNBC) cells *in vivo*. The purpose of this study is to determine how the highly metastatic TNBC cell line, MDA-MB-231, migrates in response to well-defined CXCL12 gradients *in vitro*. Traditional 2D transwell migration assays were optimized to gauge the MDA-MB-231 cells’ responsiveness to various CXCL12 concentrations. The optimum chemoattractant concentrations were applied to a commercially available 3D chemotaxis assay as stable linearly diffused gradients. Cells were embedded in type 1 bovine collagen at two different collagen concentrations, and individual unlabeled cells were monitored for 24 hours using brightfield microscopy. Time-lapse videos were used to track cell movement and shape. Quantitative data analysis was performed using an automated tracking software to measure chemotactic parameters based on cell morphology. MDA-MB-231 cells were responsive to CXCL12 concentrations greater than 200 ng/mL in 2D and 3D systems. In 3D systems, significant directed migration was observed in denser collagen matrices. It was observed that in 3D matrices a range of cell morphologies was present. Therefore, chemotaxis was evaluated as a function of cell shape revealing some differences between sub cellular populations. Our findings show the cell’s shape influences the chemotactic sensing towards CXCL12 gradients.

## Introduction

Chemotaxis, the directed migration of cells in response to a chemical stimulus, is responsible for many physiological events such as angiogenesis, wound healing, and cancer metastasis [1]. Chemotactic migration of cancer cells is initiated by the release of chemoattractants, such as chemokines and growth factors, from neighboring cells within the extracellular matrix (ECM). At the onset of metastasis, the associated cell surface receptors to the chemoattractants assist the invading primary tumor cells to sense soluble chemical gradients, to aid in directed migration into the bloodstream and proliferation at a secondary site [1,2]. For triple-negative breast cancer (TNBC) cells, where there is a lack of estrogen, progesterone, and human epidermal growth factor 2 receptors, there are a limited number of chemoattractants that could promote their migration. Previous studies have shown epidermal growth factor receptor (EGFR) and G-protein coupled receptor, CXCR4, are prevalent in the TNBC cell line, MDA-MB-231 [3–6]. CXCR4, which has only one known associated ligand, CXCL12, has been extensively studied using *in vitro* migration assays [7–10].

Transwell migration assays, a two-chamber system separated by a porous membrane, have been widely used to measure chemotaxis for two-dimensional (2D) migration. Several studies have conducted CXCL12-mediated chemotaxis of MDA-MB-231 cells with this assay in a high-throughput screening manner [3,7,11–13]. However, these previous findings have reported various cell densities, concentration ranges, and serum exposure times, making it challenging for comparative analysis [14]. Although 2D migration assays have brought fundamental insight into TNBC cell migration, it does not reflect how these cells behave in a three-dimensional (3D) ECM environment [15]. Modern-day *in vitro* chemotaxis assays have adapted to 3D cell culture systems, where TNBC cells are embedded in a 3D matrix, primarily made of type 1 collagen, and exposed to a linearly diffused gradient [16–19]. In 2013, Kim et al. discovered MDA-MB-231 cells displayed chemotactic migration when exposed to CXCL12 gradients in a microfluidic system containing type 1 rat tail collagen, suggesting that CXCL12 alone does promote directed migration [20]. However, since then, new reports have shown that the physical properties of the collagen matrix play a vital role in cell migration.

Collagen stiffness, pore size, and source material aid in the cells’ ability to migrate in a 3D system [21]. Type 1 bovine collagen is an alternative material used for 3D cell culture [22]. Fibril morphology studies using confocal microscopy found the organization of collagen fibrils differs significantly between rat tail and bovine collagen, impacting MDA-MB-231 cells’ migration [23,24]. As of now, little is known about how the mechanical properties of the collagen matrix influence the chemotactic sensing of MDA-MB-231 cells to CXCL12 gradients. Furthermore, studies have shown cell shape can influence chemotaxis and taking into consideration MDA-MB-231 cells’ phenotypically heterogeneous nature, it remains to be understood how these morphological subpopulations respond to the chemokine gradient [25,26].

In this work, we investigate MDA-MB-231 cells response to several CXCL12 linear gradients (ranging from 0 to 500 ng/mL) embedded in type 1 bovine collagen matrices at two different concentrations, 1.5 and 2.0 mg/mL. Time-lapse images for 24 hours were captured and analyzed to obtain information on the dependence of cell shape on migration patterns [27].

## Materials and methods

### Materials

Human recombinant CXCL12 protein was purchased from Fujifilm Irvine Scientific. MDA-MB-231 cells were obtained from ATCC (catalog no. HTB-26™). Dulbecco’s’ modified eagle medium (DMEM) powder and L-glutamine were purchased from Caisson Labs. Penicillin-streptomycin solution was purchased from Cytiva, and fetal bovine serum was obtained from Avantor. DMEM media was prepared by the UIUC Cell Media Facility. QCMTM Chemotaxis 96-Well Cell Migration Assay (pore size 8 µm) were purchased from Millipore Sigma. Type I collagen (bovine, 5 mg/mL), 10x DMEM with phenol red, and chemotaxis μ-slides were purchased from Ibidi.

### Cell culture

MDA-MB-231 cells were maintained in DMEM supplemented with 10% FBS, 4 mM L-glutamine, and 1% penicillin-streptomycin. The cells were grown in 182 cm^2^ cell culture flasks and placed in a humidified incubator at 37 °C at 5% CO_2_. The cell culture media was changed every 2–3 days. Cells were grown up to 70–90% confluency before they were cultured for chemotaxis assays. Cells from passage 5–20 were used for chemotaxis experiments.

### Transwell assay

The procedure was adapted from the manufacturer’s protocol. Cells were resuspended in serum free DMEM to have a final concentration of 750,000 cells/mL. 100 µL of the cell solution was placed in the upper chamber and 150 µL of serum free media in the lower chamber. The cells were starved for 18–24 hours in the incubator at 37°C, 5% CO_2_. The following day, the media in the lower chamber was replaced with 150 µL of chemoattractant media. After a 48-hour incubation at 37°C, the upper chamber was transferred to a new lower chamber with cell detachment buffer to displace the cells from the porous membrane (24-hour incubation results are provided in S3 Fig). After 30 minutes, the transwell plate was removed from the incubator, lightly tilted back and forth to ensure the cells were completely detached. After, 50 µL of a 4X Lysis buffer/CyQuant GR Dye was added to the detachment buffer to lyse the cells and label their nucleic acids with a fluorescent dye. The mixture of each well was transferred to a plate suitable for fluorescence measurement and read on a plate reader at an excitation of 480 nm and emission of 520 nm. The negative control consisted of cells with no chemoattractant and the positive control included cells exposed to CXCL12. Measurements were performed in three technical replicates. Normalized fluorescence was calculated as the ratio of the positive control to the negative control.

### 3D chemotaxis assay

The procedure for creating the collagen matrix was adapted from Ibidi Application Note 26 [28]. Experiments were done on multiple batches of cells over a period of 2 years. Chemotaxis µ-slides were placed inside petri dishes with wet kimtech wipes to maintain humidity. Small aliquots of DMEM with and without FBS were placed in the incubator at 37°C overnight. The day of the experiment, the cells were washed twice with 1x phosphate buffer saline and detached from the flask using 0.25% EDTA-trypsin buffer. The cell pellet was resuspended in serum-free DMEM (SF DMEM) at a final concentration of 12 million cells/mL. The reagents for the collagen gel mixture were placed on wet ice prior to mixing. The reagents were added based on the order of addition in Table 1, with excessive mixing in between each step to ensure homogenous distribution of collagen and cells. 6.5 µL of the collagen mixture was added to ports A and B (main channel) and plugged gently with tweezers. For gel polymerization, the slides were placed in the incubator for 1 hour at 37°C. After, 65 µL of SF DMEM was added to the reservoirs on both sides of the main channel and 0–500 ng/mL of CXCL12 was added to the left reservoir in 15 µL increments to form a gradient. All experimental conditions were repeated at least three times. Cell movement was monitored on a Zeiss Axio Z1 Observer microscope with a 37 °C, 5% CO_2_ humidified heat chamber. Using bright-field microscopy, timelapse images were taken every 10 min for 24 hours.

**Table 1:** Order of addition for collagen gel mixture.

Reagents	1.5 mg/mL collagen (µL)	2.0 mg/mL collagen (µL)
10x DMEM	20	20
1 M NaOH	11.5	15
H_2_O	46	5
7.5% NaHCO_3_	10	10
DMEM with FBS	50	50
4 mg/mL type 1 bovine collagen	112.5	150
12x10^6^ cells/mL	50	50

### Data acquisition and statistical analysis

Timelapse images were analyzed using the automated tracking ImageJ software, CellTraxx [27]. Software version 4.8 is available on GitHub (https://github.com/borge-holme/celltraxx_download). CellTraxx pixel settings were adjusted for the Zeiss microscope. The 145-time frame videos were inverted for the cells to be identifiable with a dark outline prior to tracking. Cells that fit within the tracking parameters listed in Table 2 were included in the data analysis. Cells that move for less than 6 hours were not included in the tracking analysis. Additional tracking parameters can be found in the (S1 Dataset). At least 50 cells were tracked per replicate for each condition.

**Table 2:** Significant parameters for single cell analysis using CellTraxx.

Parameter	
Pixel Size	0.7206 µm
Smallest cell Diameter	3 µm
Largest cell Diameter	50 µm
Cutting cell diameter	1 µm
Time between Images	10 minutes
Highest cell velocity	5 µm /min
Shortest cell Track	36 images (~6h)

The forward migration index (FMI), which is the efficiency of the cell’s forward migration, and center of mass (COM), representing the average of single cell endpoints, were calculated to measure chemotaxis. Both FMI and COM were calculated parallel to the gradient, (x-direction), and perpendicular to the gradient (y-direction) where n is the total number of individual cells, x_i,end_ and y_i,end_ are the cell’s endpoint coordinates, and d_i,accum_ is the total distance the cell has traveled [29].


$$\begin{array}{c} \begin{array}{c} {FMI}_{II}=\frac{\text{1}}{n}\sum_{i=\text{1}}^{n}\frac{x_{i,end}}{d_{i,accum}} \end{array} \end{array}$$ (1)

$$\begin{array}{c} \begin{array}{c} {FMI}_{⊥}=\frac{\text{1}}{n}\sum_{i=\text{1}}^{n}\frac{y_{i,end}}{d_{i,accum}} \end{array} \end{array}$$ (2)

$$\begin{array}{c} \begin{array}{c} COM=\frac{\text{1}}{n}\sum_{i=\text{1}}^{n}\left(x_{i,end},y_{i,end}\right) \end{array} \end{array}$$ (3)

Significant chemotactic activity was determined if (1) FMI_II_ was greater than the FMI_⊥_ and (2) FMI value of the CXCL12 gradient was larger than the negative control. Cell velocity was measured to monitor changes in cell movement. For statistical analysis, an ordinary one-way analysis of variance (ANOVA) test was performed followed by Dunnett’s multiple comparison test. Figure plots and statistical analysis were plotted using GraphPad Prism 9.

## Results and discussion

### CXCL12 promotes chemotactic activity of TNBC cells in a 2D transwell assay

**Fig 1:**
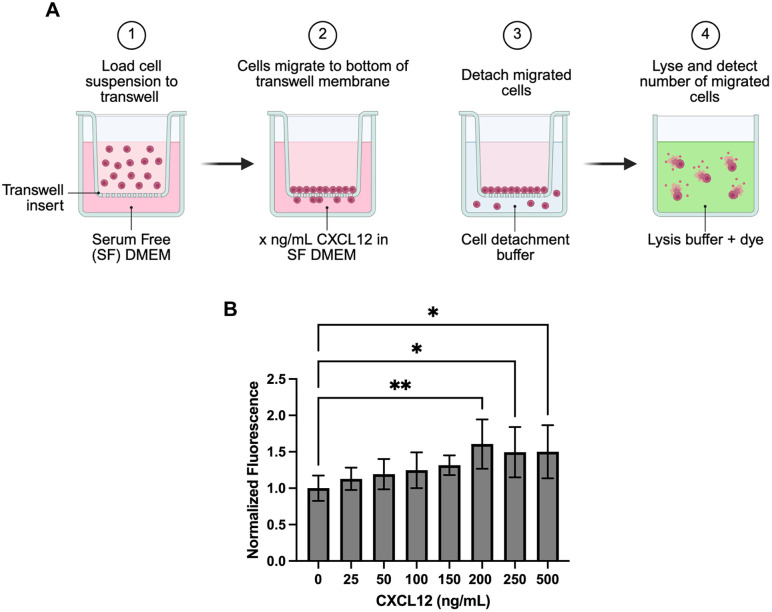
2D migration setup and migration analysis. (A) Schematic of 2D transwell migration assay made in BioRender and (B) chemotactic response of MDA-MB-231 cells to seven different CXCL12 gradients ranging from 0 to 500 ng/mL. 7.5x10^4^ cells/well migrated towards the lower chamber for 48 hours. Fluorescence measurements were normalized to the negative control. Error bars are represented as standard deviation (n = 3).*p < 0.05,**p < 0.01, Dunnett’s multiple comparison test.

An initial assessment of the chemotactic effects of CXCL12 gradients on MDA-MB-231 cells was conducted using a transwell assay with a fluorometric readout for quantitative analysis (Fig 1A). Several concentrations of CXCL12 were tested, ranging from 0 to 500 ng/mL, over a 48-hour time period in a serum free environment to enhance the cells’ sensitivity to the chemokine gradient. In Fig 1B, the number of cells migrating into the lower chamber increased as the concentration of CXCL12 increased, with a significant migration occurring between 200–500 ng/mL and the highest chemotactic activity occurring at 200 ng/mL. This optimal concentration range is similar to previously reported results in other 2D migration assays [30]. Overall, the assay provided insight into the migratory response of the TNBC cells to the chemoattractant. However, there are significant drawbacks to utilizing this assay, particularly the inability to measure the cell’s motility in real time and the formation of a steep gradient, which is not comparable to the gradients formed *in vivo,* and the instability of the gradient over time [31]. Therefore, the concentrations of CXCL12 yielding the most cell migration in this 2D assay were applied to a 3D cell culture system as linearly diffused gradients.

### Tracking individual cells in a 3D matrix using CellTraxx

**Fig 2:**
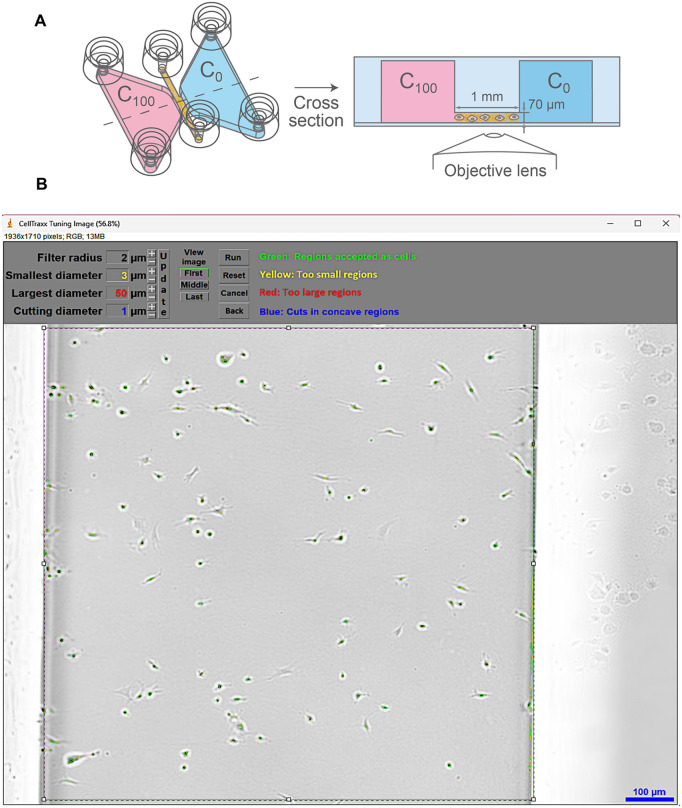
3D migration setup and cell tracking. (A) Diagram of the Ibidi µ-slide for chemotaxis (Image courtesy of Ibidi GmbH) (B) Snapshot of CellTraxx tuning interface. Zeiss microscope images were inverted to make the cells outline detectable by the CellTraxx software. The formatted video was uploaded to CellTraxx version 4.8. The CellTraxx settings were adjusted to accurately identify MDA-MB-231 cells shape and movement.

3D migration was observed using the Ibidi µ-slide for chemotaxis (Fig 2A). This device establishes a long-term linear gradient that can diffuse through a 3D matrix and monitor individual cells’ migration and morphology over time [32]. Gradient stability was verified across several studies by measuring the intensity of fluorophores with molecular weights similar to CXCL12, and a linear gradient lasting over 48 hours was observed across the migration arena [22,32,33]. Type 1 bovine collagen was used as the 3D matrix to mimic the ECM. Prior studies have shown collagen matrices from the same source material at different concentrations, ranging from 0.8 to 3.0 mg/mL, can impact breast cancer cell invasion due to their mechanical properties [23,34]. Among these studies, the range of 1.5 to 2.0 mg/mL was commonly reported for chemotaxis assays [22,35]. Therefore, we tested these two collagen concentrations to determine how a small increase in collagen density could impact cell behavior in the presence of a chemoattractant gradient.

MDA-MB-231 cells are known for their cellular heterogeneity and dynamic movement, making it difficult and time-consuming to monitor these cells using manual tracking [26,36,37]. Thus, to accurately identify and track these cells for 24 hours, the automated software CellTraxx was used as a macro plugin in ImageJ. Time-lapse images were uploaded to CellTraxx version 4.8 to measure the cell’s aspect ratio in addition to other migration parameters (Fig 2B). Extensive optimization was conducted for the CellTraxx settings “highest cell velocity” and “time between images” to ensure an accurate track of an individual cell’s movement. Additional CellTraxx parameters, particularly the Gaussian filter radius, pixel size, and gray threshold, were adjusted to accurately identify the cell outline within the Zeiss microscope settings (S1 Dataset).

**Fig 3:**
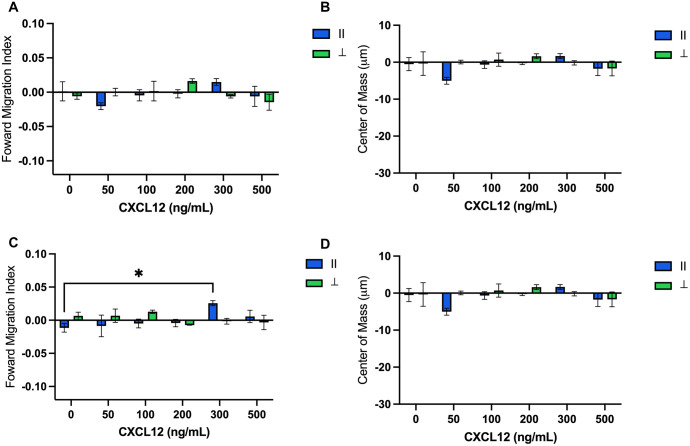
FMI and COM of the entire cell population exposed to several CXCL12 gradients. (A) Forward Migration Index and (B) center of mass of the entire MDA-MB-231 cell population in the presence of a CXCL12 linear gradients in 1.5 mg/mL collagen gel. (C) and (D) FMI and COM in 2.0 mg/mL conditions. Blue bars represent FMI and COM parallel (∥) to the gradient and green bars represent the perpendicular (⟂) to the gradient. *p < 0.05, Dunnett’s test. Error bars represented as error of mean.

Initial cell tracking was conducted on the entire cell population in the 1.5 and 2.0 mg/mL collagen concentrations. Globally, for the cells embedded in the 1.5 mg/mL collagen gel in the presence of no chemoattractant, the FMI_II_ is 0.001 ± 0.014 and FMI_⊥_ is −0.006±0.004 (Fig 3A). The COM_II_ and COM_⊥_ are −0.508± 1.774 µm and −0.366± 3.179 µm, respectively as shown in Fig 3B. Surprisingly, small amounts of CXCL12, about 50 ng/mL, induced a strong FMI_II_ in the negative direction with a value of −0.020±0.005. The COM_II_ and COM_⊥_ are −5.018±0.947 µm and −0.045±0.509 µm, respectively (Figs 3A and 3B). As the concentration of CXCL12 increased beyond 50 ng/mL, FMI_II_ progressively increased in a dose-dependent manner until reaching 300 ng/mL (S1 Fig). The chemotactic response reaches a maximal level at approximately 300 ng/mL CXCL12; the FMI_II_ value of 0.015± 0.005 and FMI_⊥_ of −0.006± 0.002, fits the first criteria of chemotaxis. The COM_II_ and COM_⊥_ are 1.668± 0.638 µm and −0.159± 0.581µm, respectively. At 500 ng/mL CXCL12, the FMI_II_ decreased, possibly indicating a lack of cellular response due to potential gradient saturation. The FMI_⊥_ behaved similarly to the FMI_II_ until 200ng/mL and then declining at 300 and 500 ng/mL, a consistent pattern also observed in the COM. We note that the FMI values are quite small, and it is definitely peculiar that the cells moved backwards at 50 ng/mL CXCL12 in this assay, which might suggest that at this concentration, CXCL12 can act as a chemorepellent in low-density matrices; it is also peculiar that the perpendicular values for FMI and COM were not always zero. This latter result suggests that the chemical gradient may not have been strictly formed in the x-direction, in spite of our many efforts to ensure this [38].

For 2.0 mg/ml collagen concentration conditions, the FMI_II_ is −0.011± 0.007 and FMI_⊥_ is −0.007± 0.006 when there is no chemoattractant (Fig 3C, S1 Movie). The COM_II_ and COM_⊥_ are −1.304± 0.652 µm and  .0707± 0.986 µm, respectively (Fig 3D). The FMI_II_ and COM_II_ have a relatively more negative value in comparison to the 1.5 mg/mL collagen negative control, which can be attributed to the increase in collagen density. However, the trends are similar in both conditions (S1 Fig). It was observed 300 ng/mL significantly promoted the forward migration of the TNBC cells with a FMI_II_ value of 0.026±0.004 and FMI_⊥_ is −0.007±0.006 (S2 Movie). The COM_II_ and COM_⊥_ are 3.013± 0.967µm and −1.014± 1.231µm, accordingly. A tighter standard deviation was noticed across all conditions, in comparison to the 1.5 mg/mL collagen matrix. The apparent “backwards” cell movement at ~50 ng/mL CXCL12 for the 1.5 mg/mL collagen matrix was not evident at 2.0 mg/mL collagen concentration, suggesting that apparent chemorepellent behavior of a nominal chemoattractant is ECM-dependent. Overall, cell migration of these triple-negative breast cancer cells was rather modest, with only 300 ng/mL CXCL12 at a collagen density of 2.0 mg/mL showing statistical significance. A recent study found that TNBC cells on stiffer substrates show increased CXCR4 expression and could provide some context as to why more chemotactic activity was observed in the denser matrix [39]. To validate the functionality of the migration assay with this cell line, we tested another well-established chemoattractant, epidermal growth factor (EGF). The cells exhibited positive migration toward both 10 ng/mL and 100 ng/mL EGF gradients (S4 Fig), reinforcing that the trends observed with CXCL12 reflect genuine cell behavior rather than artifacts of the assay setup.

### MDA-MB-231 cells response to CXCL12 is shape dependent

One of the initial steps of TNBC metastasis involves extensive cytoskeletal reorganization, where metastatic cells transition from a rounded epithelial-like shape to an elongated, mesenchymal-like shape [40]. This process enables the cells to detach from the primary tumor, intravasate into the vasculature, and proliferate at a secondary site [41]. It is well-known that the CXCL12/CXCR4 axis contributes to migratory and invasive phenotypes in breast cancer cells [42,43]. However, it is yet to be understood how the cell morphologies within the heterogeneous population respond to the chemoattractant gradient.

**Fig 4:**
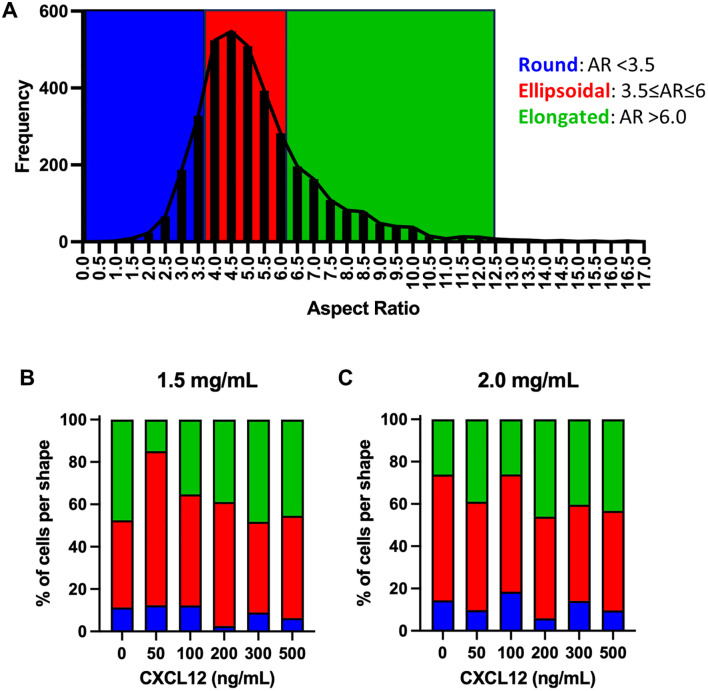
Distribution of different cell shapes. (A) Histogram plot of the aspect ratio (AR) of all single cells. Cell shape was divided into three AR ranges: round (AR less than 3.5), ellipsoidal (AR between 3.5 and 6.0), and elongated (AR more than 6.0). (B) Percentage of each cell shape population for 1.5 mg/mL and (C) 2.0 mg/mL type 1 bovine collagen conditions derived from the histogram. Blue bars represent round cells, red bars represent ellipsoidal cells, and green bars represent elongated cells.

Based on the initial assessment of the entire cell population, we hypothesize that the cell’s morphology may be contributing to the large deviations of migration behavior found across the tested gradients. To test this hypothesis, the average aspect ratio (AR) for each cell was calculated, plotted on histogram plot and assigned a cell shape: round, ellipsoidal, and elongated (Fig 4A) [44,45]. The median of the histogram plot, AR between 3.5 and 6.0, was assigned as the ellipsoidal due to the cell’s constant retraction and contraction behavior. Cell with an AR less than 3.5 was classified as round, where the cells remain in the round shape throughout the duration of the experiment. Cells with AR more than 6.0 were classified as elongated, due to the cell’s elongation morphology within the collagen matrix. As shown in Fig 4B, there is a difference in the cell shape population within the two different collagen matrices for each gradient. With no chemoattractant present, the percentage of round cells remained similar for both collagen matrices, a bulk of cells were ellipsoidal at the 2.0 mg/mL, and the majority were elongated in the 1.5 mg/mL collagen gel. Although the percentages for the ellipsoidal cell shape remained similar for each gradient, there was a noticeable difference for the round and elongated cells when exposed to the 300 ng/mL CXCL2 gradient, where there was an increase in the round cells at 2 mg/mL collagen and a decrease in the 1.5 mg/mL collagen conditions (S1 Table). After the classification of cell shapes, the chemotaxis data was subsequently re-analyzed, and averages were calculated for each group. Since the gradient was established in the x-direction, only the FMI_II_ and COM_II_ are highlighted in this section for the different cell shapes (denoted as FMIx and COMx). Cell velocity was also included in our measurements to determine if cell migration speed differs between cell shapes.

#### Migratory behavior of round cells.

**Fig 5:**
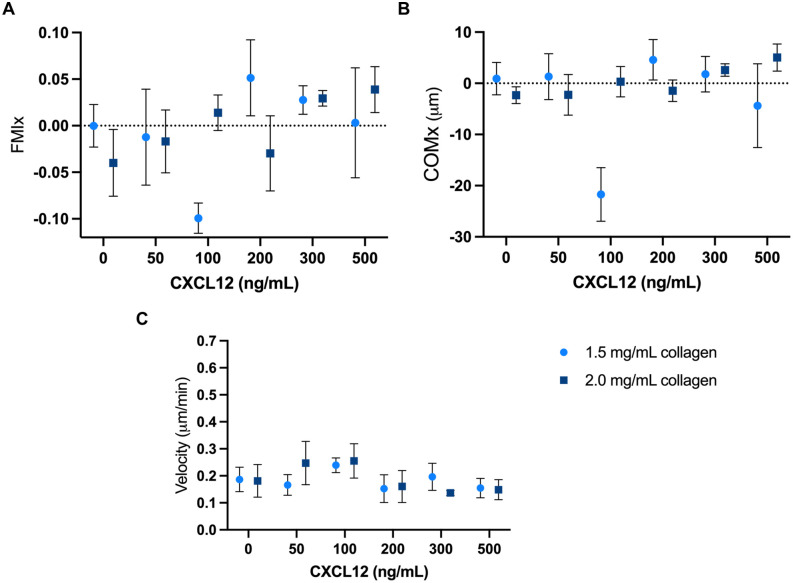
Migration analysis of round cells. (A) FMI, (B) COM, and (C) Velocity of the round shaped MDA-MB-231 cells in the presence of a CXCL12 linear gradients. Error bars represent standard error of mean.

The round cells display different migratory patterns in the two collagen matrices despite being exposed to the same CXCL12 gradients. In the absence of CXCL12, cells in the 2.0 mg/mL collagen matrix showed more negative FMI and COM values compared to those in the 1.5 mg/mL matrix (Fig 5A, 5B). In the 1.5 mg/mL matrix, cells displayed a negative FMI across 0–100 ng/mL CXCL12, then changed to a positive FMI between 200 and 500 ng/mL. The highest FMI in this matrix occurred at 200 ng/mL, reaching 0.051 ± 0.041. In the denser collagen matrix, a more consistent upward trend in FMI was observed with increasing CXCL12 concentration, except at 200 ng/mL. The peak migratory response in the denser matrix was recorded at 500 ng/mL, with an FMI of 0.039 ± 0.025. Cell velocities were comparable between the two matrix conditions for most CXCL12 concentrations, with the exception of the low 50 ng/mL and high 300 ng/mL concentrations (Fig 5C). In comparison to the ellipsoidal and mesenchymal cells, the round cells have a relatively higher COM value when exposed to high CXCL12 concentrations, suggesting that the cells spend less time interacting with the ECM (S5 Fig). Interestingly, the velocity decreases when the round cells are exposed to CXCL12 gradients >200 ng/mL and migrate slower in comparison to when there is no chemoattractant present. Additionally, the averaged velocities of the round cells are less than the reported values of the ellipsoidal and elongated cells, which contradicts previous works that suggest cells exhibiting an ameboid migration move relatively faster [19].

#### Migratory behavior of ellipsoidal cells.

**Fig 6:**
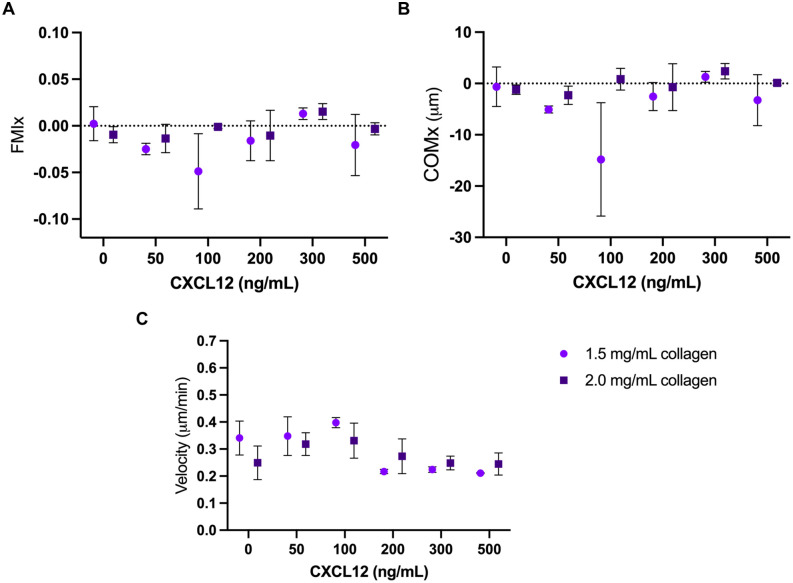
Migration analysis of ellipsoidal cells. (A) FMI, (B) COM, and (C) Velocity of the ellipsoidal shape MDA-MB-231 cells in the presence of a CXCL12 linear gradients. Error bars represent standard error of mean.

This group demonstrated the most random migration, where the cells are almost non-responsive to any of the CXCL12 gradients in both collagen matrices (Fig 6A). In the 1.5 mg/mL collagen, the FMI began to decrease in the negative direction from 0 to 100 ng/mL, then increased in the positive direction up to 300 ng/mL. In the 2.0 mg/mL collagen, less variability in the SEM was observed amongst the replicates, indicating the cells migrate in a consistent behavior within each concentration. For both collagen matrices, the highest FMI value for this subpopulation was observed at 300 ng/mL: 0.013 ± 0.006 in 1.5 mg/mL and 0.015 ± 0.009 in 2.0 mg/mL. There was little to no change in the COM for each concentration, except for 100 ng/mL in the 1.5 mg/mL collagen (Fig 6B). For both matrices, these cells had an overall higher velocity from 0 to 100 ng/mL and then migrated more slowly when exposed to gradients from 200 to 500 ng/mL (Fig 6C). In the 1.5 mg/mL collagen, average velocities were consistent across biological replicates from 200 to 300 ng/mL, whereas in the 2.0 mg/mL collagen, they were consistent from 300 to 500 ng/mL. Interestingly, there is a switch in migratory behavior where the ellipsoidal cells in the denser collagen matrix migrate slightly faster towards the high CXCL12 gradients (S1D Table). Additionally, we noticed the velocity of this subpopulation behaves similarly to the round cells when a gradient of  ≥ 200 ng/mL was introduced (S5 Fig).

#### Migratory behavior of elongated cells.

**Fig 7:**
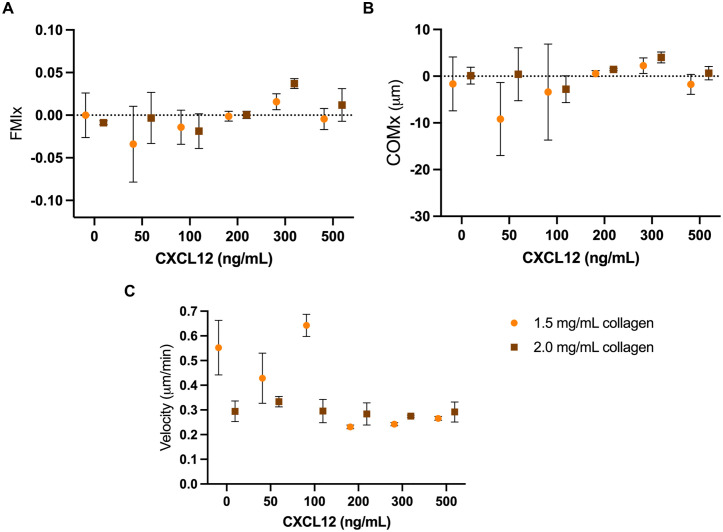
Migration analysis of elongated cells. (A) FMI, (B) COM and (C) Velocity of the elongated shape MDA-MB-231 cells in the presence of a CXCL12 linear gradients. Error bars represent standard error of mean.

Between 0–50 ng/mL, the cells have large fluctuations in their FMI in the 1.5 mg/mL collagen matrix than the 2.0 mg/mL collagen matrix. The elongated cells seem to favor the denser matrix with a 300 ng/mL chemokine gradient as denoted by the FMI value of 0.037, which is the largest value observed across all the conditions (Fig 7A). As the averaged distance traveled of the elongated cells increased towards the high CXCL12 gradients, similar COM values were measured at CXCL12 concentrations of 300 ng/mL in both collagen matrices. As the CXCL12 concentration increases, we observed smaller standard deviations across the biological replicates, indicating this is consistent migratory behavior for the cells (Fig 7B). When the cells interacted with the low CXCL12 gradients, between 0–100 ng/mL, there was a noticeable difference in cell velocities, where the cells migrate faster in the 1.5 mg/mL than in the 2.0 mg/mL collagen gel (Fig 7C). For the high CXCL12 gradients, between 300–500 ng/mL, the reported average velocities are identical in both matrices, calculated to be in a range of 0.2 to 0.3 µm/min. In the denser matrix, the cell velocities remain the same across all gradients, suggesting the cells are strongly interacting with the ECM while simultaneously sensing the chemoattractant.

## Conclusion

In this work, we explored the relationship between cell shape and chemotactic cell migration in a type 1 bovine collagen matrix. As CXCL12 is established as a chemoattractant for breast cancer cells, as confirmed by our 2D transwell assay, it was a surprising observation to see CXCL12 by itself was not a potent chemoattractant in our 3D system. In both collagen gel concentrations, the cells exhibited an overall random migration when exposed to CXCL12 gradients. When cells were categorized based on their shape and had the same CXCL12 gradient applied, we found there were larger fluctuations in the matrices for the round cells in comparison to the ellipsoidal and elongated cells. Additionally, we found the round and ellipsoidal cells did not have a change in the average FMIx in the denser matrix, but the average FMIx of the elongated cells increased over two-fold in the denser matrix when exposed to a 300 ng/mL CXCL12 gradient.

Our findings suggest the need to explore chemotaxis in heterogeneous TNBC cells using complex 3D ECM mimics. The limited response of MDA-MB-231 cells to CXCL12 gradients implies these cells may require added mechanical and chemical cues common in physiological environments to assist with directed migration. For example, collagen fiber alignment parallel to the gradient can help cells migrate farther toward the chemoattractant [46]. Additionally, conflicting studies have shown that uniform EGF either promotes or nullifies migration to CXCL12 gradients [20,47]. Applying these additional parameters could give us a better fundamental understanding of how chemotaxis plays a role in TNBC metastasis.

## Supporting information

S1 Table Tabulated results of migratory analyses for 1.5 mg/mL and 2.0 mg/mL collagen matrix conditions. (DOCX)

S1 Fig Representative trajectory plots of MDA-MB-231 cells embedded in 1.5 mg/mL collagen matrix. (A) 0 ng/mL (B) 50 ng/mL (C) 100 ng/mL (D) 200 ng/mL (E) 300 ng/mL (F) 500 ng/mL CXCL12 gradients.
(DOCX)

S2 Fig Representative trajectory plots of MDA-MB-231 cells embedded in 2.0 mg/mL collagen matrix. (A) 0 ng/mL (B) 50 ng/mL (C) 100 ng/mL (D) 200 ng/mL (E) 300 ng/mL (F) 500 ng/mL CXCL12 gradients.
(DOCX)

S3 Fig Transwell measurements of MDA-MB-231 cells exposure to varying concentrations of CXCL12. (A) 0–160 ng/mL, 24-hour incubation (B) 0–240 ng/mL, 24 hour incubation (C) 0–1000 ng/mL, 48 hour incubation.
(TIFF)

S4 Fig 3D chemotaxis measurements of EGF-mediated chemotaxis. (A) FMI and (B) COM of EGF chemotaxis in 1.5 mg/mL collagen matrix. (C) FMI and (D) COM of EGF chemotaxis in 2.0 mg/mL collagen matrix.
(TIFF)

S5 Fig Comparative migration analyses of round, ellipsoidal, and elongated cell shapes on a single plot. (A) FMI (B) COM (C) Velocity.
(TIFF)

S1 Movie Time-lapse video of MDA-MB-231 cells exposed to the negative control in 2.0 mg/mL with CellTraxx. (AVI)

S2 Movie Time-lapse video of MDA-MB-231 cells exposed to the 300 ng/mL CXCL12 gradient in 2.0 mg/mL with CellTraxx. (AVI)

S1 Dataset Additional CellTraxx parameter settings. (XLSX)
