# Supplementary material for: MDA-MB-231 cell morphology influences chemotactic sensing of CXCL12 gradients in type 1 bovine collagen matrix
Source: PLoS One. 2026 Jul 8;21(7):e0343188. doi: 10.1371/journal.pone.0343188 (PMC13345270; doi:10.1371/journal.pone.0343188)
Supplement: S1 Table — (DOCX) [file pone.0343188.s001.docx]

**S1A** Table. Percentages of cell shape within 1.5 and 2.0 mg/mL collagen matrices

|  | **Round** | | **Ellipsoidal** | | **Elongated** | |
| --- | --- | --- | --- | --- | --- | --- |
| [CXCL12] (ng/mL) | 1.5 mg/mL | 2.0 mg/mL | 1.5 mg/mL | 2.0 mg/mL | 1.5 mg/mL | 2.0 mg/mL |
| 0 | 11.37 | 14.41 | 41.11 | 59.46 | 47.52 | 26.13 |
| 50 | 12.4 | 9.84 | 72.73 | 51.23 | 14.88 | 38.93 |
| 100 | 12.25 | 18.49 | 52.45 | 55.48 | 35.29 | 26.03 |
| 200 | 2.6 | 5.93 | 58.59 | 48.05 | 38.82 | 46.02 |
| 300 | 8.96 | 14.18 | 42.86 | 45.42 | 48.19 | 40.39 |
| 500 | 6.37 | 9.66 | 48.28 | 47.1 | 45.34 | 43.24 |

**S1B** Table. FMIx values of different cell shape populations in 1.5 and 2.0 mg/mL collagen matrices

|  | **Round** | | | | **Ellipsoidal** | | | | **Elongated** | | | |
| --- | --- | --- | --- | --- | --- | --- | --- | --- | --- | --- | --- | --- |
| [CXCL12] (ng/mL) | 1.5 mg/ml | | 2.0 mg/ml | | 1.5 mg/ml | | 2.0 mg/mL | | 1.5 mg/ml | | 2.0 mg/ml | |
|  | FMIx | SEM | FMIx | SEM | FMIx | SEM | FMIx | SEM | FMIx | SEM | FMIx | SEM |
| 0 | -1.03E-04 | 0.023 | -0.040 | 0.036 | 0.002 | 0.018 | -0.009 | 0.009 | -4.70E-05 | 0.026 | -0.009 | 0.002 |
| 50 | -0.012 | 0.051 | -0.017 | 0.034 | -0.025 | 0.006 | -0.014 | 0.015 | -0.034 | 0.044 | -0.003 | 0.030 |
| 100 | -0.099 | 0.016 | 0.014 | 0.019 | -0.049 | 0.040 | -0.001 | 0.002 | -0.014 | 0.020 | -0.019 | 0.020 |
| 200 | 0.051 | 0.041 | -0.030 | 0.040 | -0.016 | 0.021 | -0.010 | 0.027 | -0.001 | 0.006 | 2.85E-04 | 0.004 |
| 300 | 0.028 | 0.015 | 0.029 | 0.008 | 0.013 | 0.006 | 0.015 | 0.009 | 0.016 | 0.009 | 0.037 | 0.006 |
| 500 | 0.003 | 0.059 | 0.039 | 0.025 | -0.021 | 0.033 | -0.003 | 0.006 | -0.004 | 0.012 | 0.012 | 0.019 |

**S1C** Table. COMx values of different cell shape populations in 1.5 and 2.0 mg/mL collagen matrices

|  | **Round** | | | | **Ellipsoidal** | | | | **Elongated** | | | |
| --- | --- | --- | --- | --- | --- | --- | --- | --- | --- | --- | --- | --- |
| [CXCL12] (ng/mL) | 1.5 mg/ml | | 2.0 mg/ml | | 1.5 mg/ml | | 2.0 mg/mL | | 1.5 mg/ml | | 2.0 mg/ml | |
|  | COMx (µm) | SEM | COMx (µm) | SEM | COMx (µm) | SEM | COMx (µm) | SEM | COMx (µm) | SEM | COMx (µm) | SEM |
| 0 | 0.91 | 3.16 | -2.31 | 1.64 | -0.64 | 3.86 | -1.19 | 0.91 | -1.65 | 5.75 | 0.11 | 1.80 |
| 50 | 1.30 | 4.48 | -2.24 | 3.97 | -5.07 | 0.67 | -2.30 | 1.78 | -9.17 | 7.81 | 0.41 | 5.67 |
| 100 | -21.72 | 5.24 | 0.32 | 2.96 | -14.80 | 11.06 | 0.83 | 2.11 | -3.40 | 10.27 | -2.79 | 2.88 |
| 200 | 4.60 | 3.94 | -1.44 | 2.11 | -2.56 | 2.73 | -0.73 | 4.56 | 0.56 | 0.63 | 1.45 | 0.26 |
| 300 | 1.79 | 3.47 | 2.60 | 1.21 | 1.29 | 1.05 | 2.38 | 1.51 | 2.25 | 1.67 | 4.02 | 1.18 |
| 500 | -4.37 | 8.18 | 5.04 | 2.65 | -3.26 | 4.97 | 0.12 | 0.44 | -1.75 | 2.13 | 0.66 | 1.43 |

**S1D** Table. Velocity (𝑽) values of different cell shape populations in 1.5 and 2.0 mg/mL collagen matrices

|  | **Round** | | | | **Ellipsoidal** | | | | **Elongated** | | | |
| --- | --- | --- | --- | --- | --- | --- | --- | --- | --- | --- | --- | --- |
| [CXCL12] (ng/mL) | 1.5 mg/ml | | 2.0 mg/ml | | 1.5 mg/ml | | 2.0 mg/mL | | 1.5 mg/ml | | 2.0 mg/ml | |
|  | 𝑽 (µm/min) | SEM | 𝑽 (µm/min) | SEM | 𝑽 (µm/min) | SEM | 𝑽 (µm/min) | SEM | 𝑽 (µm/min) | SEM | 𝑽 (µm/min) | SEM |
| 0 | 0.187 | 0.045 | 0.181 | 0.060 | 0.341 | 0.063 | 0.249 | 0.062 | 0.552 | 0.110 | 0.294 | 0.042 |
| 50 | 0.166 | 0.038 | 0.247 | 0.080 | 0.348 | 0.071 | 0.318 | 0.042 | 0.428 | 0.101 | 0.333 | 0.021 |
| 100 | 0.239 | 0.027 | 0.255 | 0.064 | 0.397 | 0.019 | 0.331 | 0.065 | 0.643 | 0.045 | 0.295 | 0.047 |
| 200 | 0.152 | 0.051 | 0.161 | 0.059 | 0.217 | 0.008 | 0.273 | 0.064 | 0.231 | 0.008 | 0.284 | 0.045 |
| 300 | 0.196 | 0.050 | 0.137 | 0.009 | 0.224 | 0.010 | 0.248 | 0.025 | 0.242 | 0.006 | 0.275 | 0.004 |
| 500 | 0.155 | 0.036 | 0.149 | 0.037 | 0.211 | 0.002 | 0.245 | 0.041 | 0.266 | 0.008 | 0.292 | 0.040 |
