# Supplementary material for: MDA-MB-231 cell morphology influences chemotactic sensing of CXCL12 gradients in type 1 bovine collagen matrix
Source: PLoS One. 2026 Jul 8;21(7):e0343188. doi: 10.1371/journal.pone.0343188 (PMC13345270; doi:10.1371/journal.pone.0343188)
Supplement: S1 Fig — (A) 0 ng/mL (B) 50 ng/mL (C) 100 ng/mL (D) 200 ng/mL (E) 300 ng/mL (F) 500 ng/mL CXCL12 gradients. (DOCX) [file pone.0343188.s002.docx]

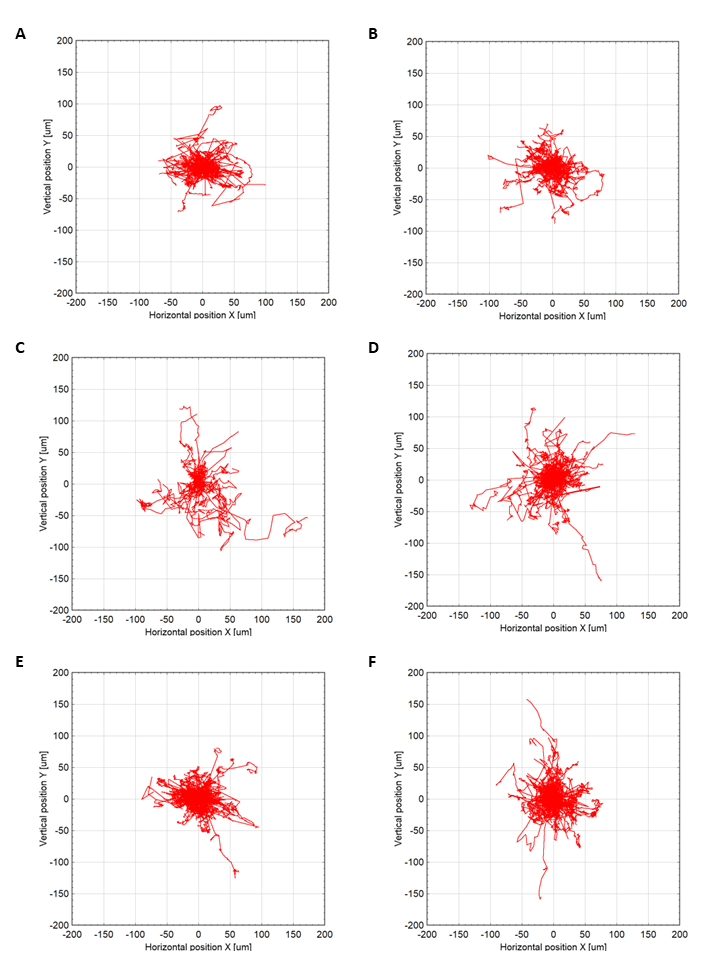


**S1 Fig**. Representative trajectory plots of MDA-MB-231 cells embedded in 1.5 mg/mL collagen matrix, tracked by CellTraxx software with a chemotactic duration ≥6 hours. (A) 0 ng/mL (B) 50 ng/mL (C) 100 ng/mL (D) 200 ng/mL (E) 300 ng/mL (F) 500 ng/mL CXCL12 gradients.
