# Supplementary figures and images for: MDA-MB-231 cell morphology influences chemotactic sensing of CXCL12 gradients in type 1 bovine collagen matrix
Source: PLoS One. 2026 Jul 8;21(7):e0343188. doi: 10.1371/journal.pone.0343188 (PMC13345270; doi:10.1371/journal.pone.0343188)

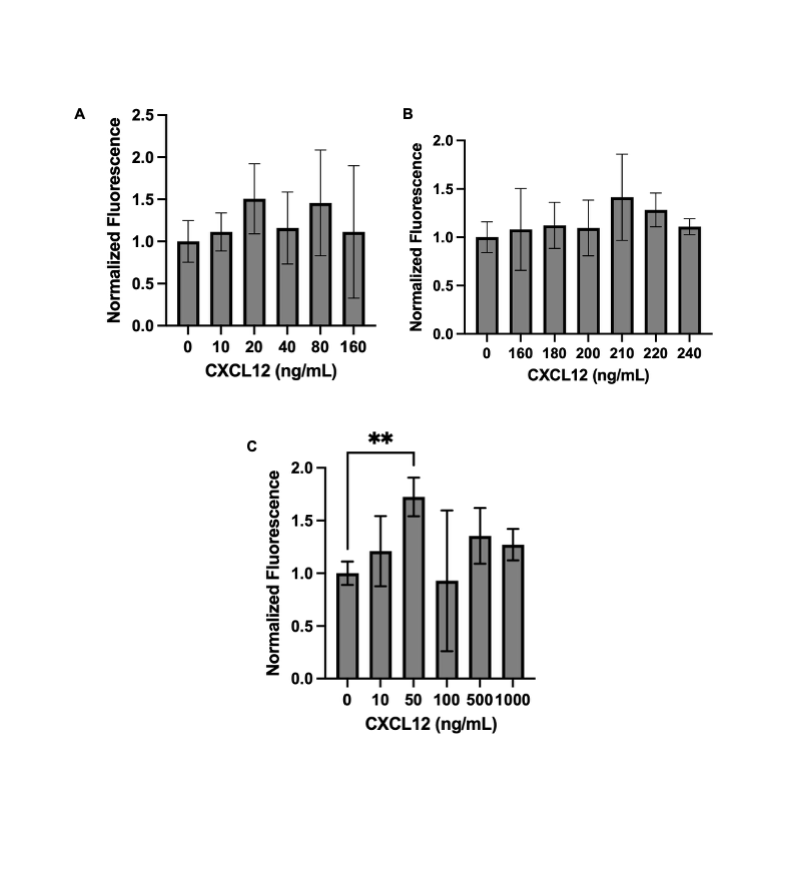

Supplement: S3 Fig — (A) 0–160 ng/mL, 24-hour incubation (B) 0–240 ng/mL, 24 hour incubation (C) 0–1000 ng/mL, 48 hour incubation. (TIFF) [file pone.0343188.s004.tiff]

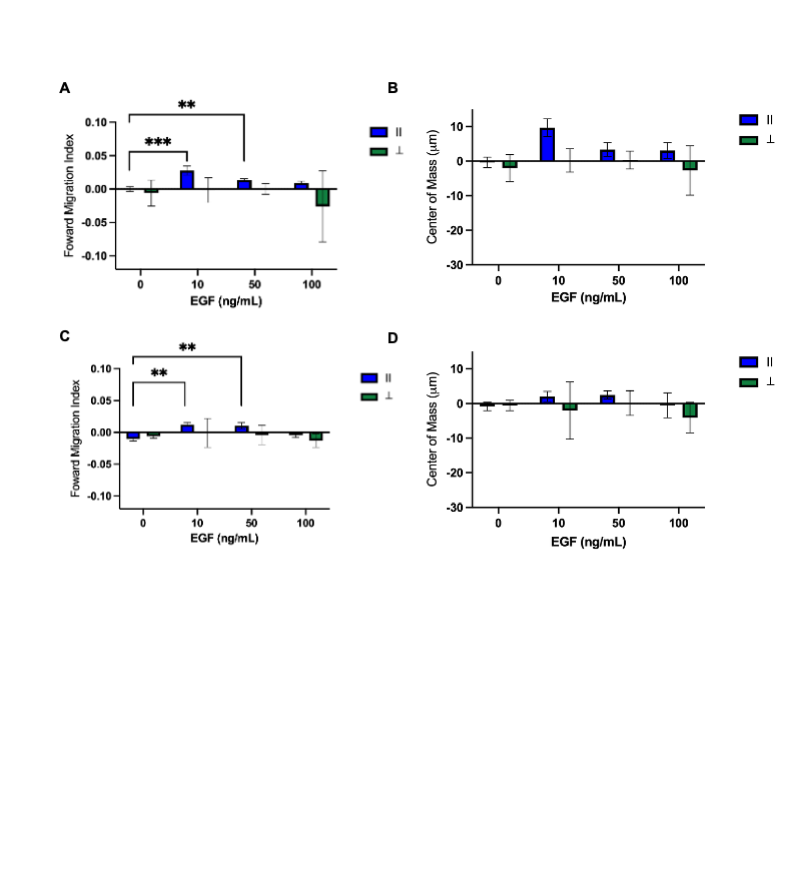

Supplement: S4 Fig — (A) FMI and (B) COM of EGF chemotaxis in 1.5 mg/mL collagen matrix. (C) FMI and (D) COM of EGF chemotaxis in 2.0 mg/mL collagen matrix. (TIFF) [file pone.0343188.s005.tiff]

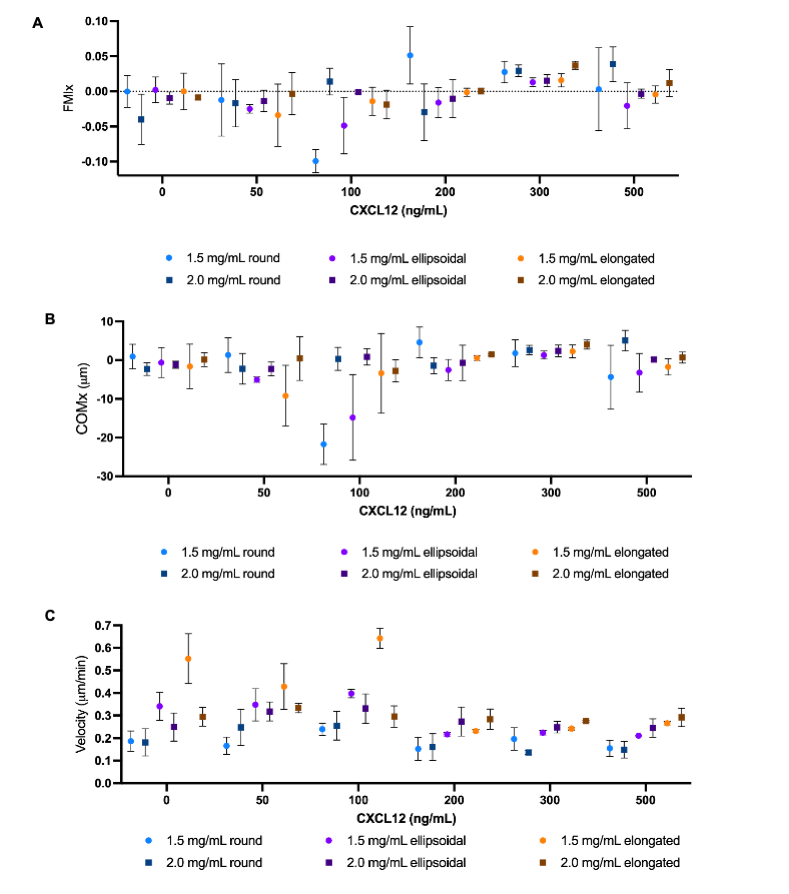

Supplement: S5 Fig — (A) FMI (B) COM (C) Velocity. (TIFF) [file pone.0343188.s006.tiff]
